# Panophthalmitis and necrotizing fasciitis following glaucoma drainage device surgery: A case report

**DOI:** 10.1016/j.ajoc.2026.102595

**Published:** 2026-05-04

**Authors:** Armaan Jaffer, Yusuf Ahmed, Georges Nassrallah, Irfan N. Kherani

**Affiliations:** a Faculty of Health Sciences, Queen's University, 20 Barrie Street, Kingston, ON, K7L 3N6, Canada; b Department of Ophthalmology & Visual Sciences, University of Toronto, 340 College Street, Suite 400, Toronto, ON, M5T 3A9, Canada; c University Health Network, 200 Elizabeth Street, Toronto, ON, M5G 2C4, Canada

**Keywords:** Glaucoma surgery, Panophthalmitis, Endophthalmitis, Necrotizing fasciitis, Glaucoma drainage device, Case report

## Abstract

**Purpose:**

Post-surgical panophthalmitis and necrotizing fasciitis are rare but potentially sight- and life-threatening complications requiring urgent treatment. We report a case of invasive Group A Streptococcus infection following combined cataract and Ahmed Glaucoma Valve implantation surgery.

**Observations:**

A 69-year-old man with advanced neovascular glaucoma, diabetes, hypertension, and atrial fibrillation underwent uneventful cataract surgery with intraocular lens placement and glaucoma drainage device implantation using a donor scleral patch. On postoperative day (POD) zero, the graft was adherent with intact conjunctival closure. By POD4, he presented with fever, confusion, orbital pain, swelling, chemosis, and purulent discharge, and was found to be in septic shock. Imaging showed exophthalmos, periocular swelling, fat stranding, and lateral rectus thickening. Examination revealed light perception vision, relative afferent pupillary defect, hypopyon, and vitreous haze. Blood and conjunctival cultures grew Group A Streptococcus. Despite intravenous and topical antibiotics, infection progressed. He underwent device explantation and subsequent enucleation with silicone implant, partial dacryoadenectomy, conformer placement, and temporary tarsorrhaphy. Orbital healing was satisfactory with resolution of infection and pain. Hospital course was complicated by aspiration pneumonia, *Clostridium difficile* colitis, and type two myocardial infarction with new heart failure. Although infection improved after a two-week course of antibiotics, the patient later suffered cardiac arrest and passed away.

**Conclusions:**

This case documents exogenous panophthalmitis and necrotizing fasciitis following glaucoma drainage device implantation caused by invasive Group A Streptococcus. The case highlights the aggressive nature of infection and underscores the importance of early recognition, aggressive management, and careful postoperative monitoring in high-risk patients.

## 1 Introduction

Although rare, post-surgical panophthalmitis and necrotizing fasciitis can be sight- and life-threatening.^1^ Urgent treatment is required because these conditions can progress rapidly and carry a poor visual and systemic prognosis.^1^ Here, we report a case of post-surgical necrotizing fasciitis and panophthalmitis following an uneventful combined cataract and Ahmed (FP7) Glaucoma Valve (AGV) (New World Medical, Rancho Cucamonga, CA) implantation surgery with a donor scleral patch.

## 2 Case report

A 69-year-old Caucasian man with advanced neovascular glaucoma and cataract in the left eye was referred to a glaucoma specialist for consideration of surgical intervention. His past ocular history was remarkable for proliferative diabetic retinopathy, previous cataract extraction and intraocular lens implantation (CE-IOL) in right eye, and radial keratotomy in both eyes. His past medical history was notable for hypertension, dyslipidemia, atrial fibrillation, sciatica, and type two diabetes mellitus of approximately 25 years’ duration, for which he had been insulin-treated for approximately 20 years. His diabetes regimen at the time of surgery consisted of metformin 1 g twice daily, insulin glargine 20 units nightly, and semaglutide 2 mg weekly. He had diabetic retinopathy and neuropathy, but no known nephropathy or macrovascular complications. He monitored glucose with a Freestyle Libre continuous glucose monitor, with typical reported readings of 5.5–6.5 mmol/L at breakfast and up to 8 mmol/L at lunch and dinner. His most recent hemoglobin A1c was 7.0%. There was no documented history of diabetic foot ulceration or other chronic soft tissue ulceration in the available chart. On initial consultation, his left eye intraocular pressure was 22 mmHg with Goldmann Applanation Tonometry despite use of four classes of topical glaucoma medication and acetazolamide 250 mg nightly. Other systemic medications included dabigatran, bisoprolol, indapamide, perindopril, pregabalin, and rosuvastatin. Best corrected visual acuity (BCVA) was counting fingers in the right eye and 20/400 in the left eye.

The patient underwent uncomplicated left eye cataract and glaucoma surgery with SA60AT 22.0D intraocular lens (IOL) and supertemporal AGV with donor scleral patch. The surgery was performed by a glaucoma specialist with fellowship training in Glaucoma and Advanced Anterior Segment Surgery. On post-operative day (POD) zero examination, the tube shunt was covered, and the scleral graft was appropriately adherent. The conjunctival closure and sutures were stable and intact. There were no appreciable signs of intraocular infection. Routine postoperative therapy included moxifloxacin (0.5%) four times daily, prednisolone acetate (1%) every 2 h while awake and bromfenac (0.07%) once daily in the surgical eye was initiated. Bromfenac was selected for its once-daily dosing to improve compliance, given the patient's complex multi-drop regimen.

**Fig. 1:**
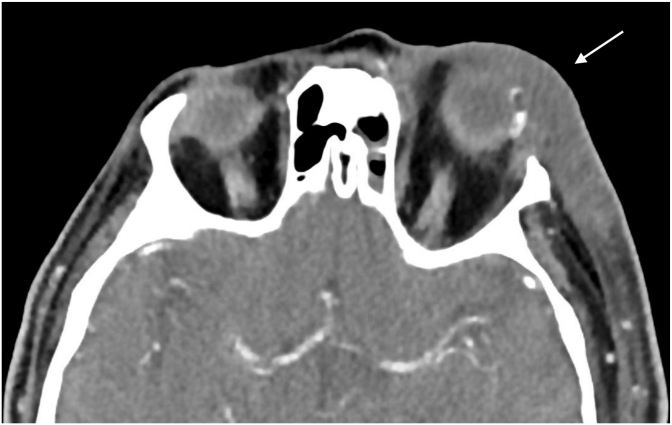
Axial contrast-enhanced CT of the orbits demonstrating marked left periorbital soft tissue swelling with fat stranding. There is evidence of exophthalmos of the left globe and a taut optic nerve.

On POD4, the patient presented to his local emergency department with a two-day history of fever and confusion with left orbital pain, swelling, proptosis, ptosis, chemosis, and purulent discharge. He was hypotensive, tachycardic and febrile with temperature of 40.1 °C. Bloodwork revealed a leukocytosis with white blood cell count of 13.25 x 10^9^/L, absolute neutrophil count of 12.33 x 10^9^/L, and lactate of 7.1 mmol/L. Computed tomography (CT) of the head without contrast (Fig. 1) revealed left exophthalmos with hyperdense material in the orbit, significant left periocular swelling, fat stranding, taut optic nerve, and left lateral rectus thickening. He was given intravenous (IV) fluids with empiric ceftriaxone 2 g, vancomycin 1.25 g, and piperacillin-tazobactam 2 g once. On the same day examination by ophthalmology, left eye BCVA was light perception (LP), a left relative afferent pupillary defect was present and significant extraocular movements limitation was noted. At this time, the conjunctival closure still appeared intact. There was a 2-mm serosanguinous hypopyon and severe vitreous haze prevented view of the posterior pole. A possible choroidal detachment was noted on bedside ultrasound.

He was transferred urgently to the intensive care unit (ICU) for intubation and systemic management in the context of panophthalmitis with septic shock. Hourly topical fortified tobramycin (13.6 mg/ml) and vancomycin (50 mg/ml) drops were added hourly to the left eye. The infectious disease team was consulted to optimize his antibiotic coverage. Conjunctival swab and blood cultures were positive for Group A Streptococcus (GAS). A retina specialist advised against bedside intravitreal injection because the patient was critically ill, intubated in the ICU, and had poor visualization with severe vitreous haze. Given the patient's hemodynamic instability, septic shock, and rapidly progressive extraocular infection, priority was placed on systemic stabilization and subsequent surgical source control.

**Fig. 2:**
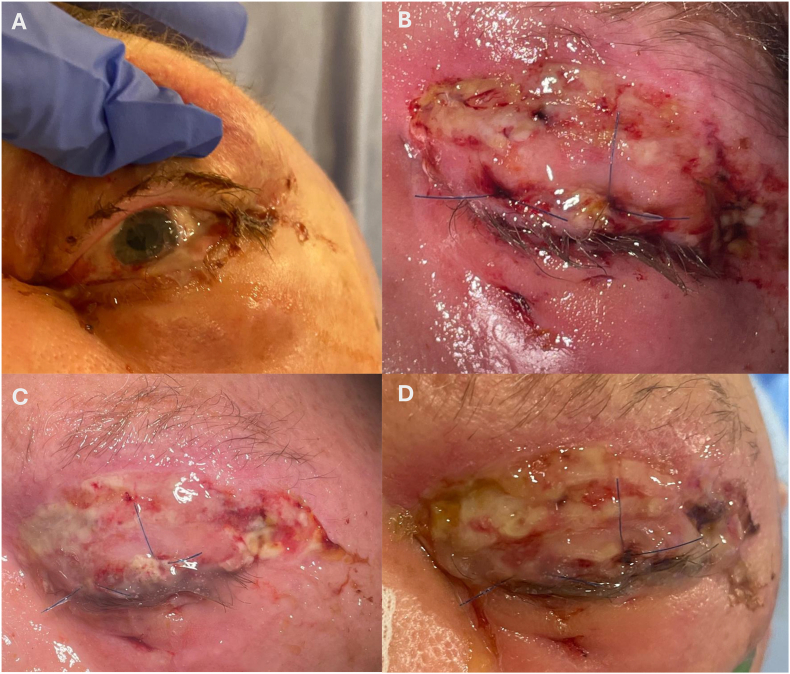
Clinical photographs demonstrating the course of iGAS panophthalmitis following combined cataract and glaucoma surgery on A) Postoperative day 0 (POD0) tube explantation; B) POD0 enucleation with soft tissue debridement with temporary tarsorrhaphy; C) POD5 enucleation showing improved swelling and inflammation and D) POW1 enucleation with infection appearing controlled with signs of early healing.

The patient returned to the operating room three days after hospital admission for tube and plate explantation. Intraoperatively, the scleral patch was found to be non-adherent to the globe with significant retraction of the conjunctiva. The invasive Group A Streptococcus (iGAS) infection continued to extend, with progressive involvement of the periorbital tissues (Fig. 2A). The oculoplastics team was emergently consulted and enucleation was performed one week after tube explantation for infection control. A silicone implant was placed and the extraocular muscles were sutured into the socket fornices with a myoconjunctival approach obviating the need for a vicryl mesh which was felt to represent a potential future nidus of infection.^2^ A partial dacryoadenectomy was also performed to remove a necrotic appearing lacrimal gland. A plastic conformer was placed in the socket. The left upper eyelid was necrotic and the anterior lamella was fully debrided along with surrounding necrotic periorbital soft tissue. The procedure was tolerated well and a complete temporary tarsorrhaphy closed the left eye (Fig. 2B).

Over the two weeks, the patient's iGAS infection appeared to regress and there was satisfactory healing of the orbital socket post-enucleation surgery, no purulent discharge, and significant reduction of inflammation and pain (Fig. 2C and D). While his local infection improved and vasopressors were stopped, the patient developed several complications: aspiration pneumonia caused *by Escherichia coli* identified through sputum culture requiring repeat intubation; *Clostridium difficile* colitis; and a type two myocardial infarction with new heart failure. After completing a two-week course of amoxicillin, the patient was clinically stable and transferred to a community hospital closer to home. However, four days after repatriation to the community hospital's ICU, the patient suffered a cardiac arrest and passed away.

## 3 Discussion

Infections following glaucoma drainage device (GDD) surgery are uncommon.^3^ A retrospective review of 542 eyes over nearly 10 years reported endophthalmitis rates of 1.7% following AGV implantation, with a fivefold higher likelihood in children compared to adults and the majority of cases (88.9%) being delayed in onset.^4^

Exogenous panophthalmitis is a rare, fulminant infection that rapidly involves every ocular layer, including the vitreous, retina, cornea, and sclera with extension into the orbital structures.^1^^,^^5^, ^6^, ^7^ Given its grave prognosis, immediate broad-spectrum systemic and intravitreal antibiotics are essential, and early enucleation or evisceration should be considered when visual potential is minimal or systemic spread is a concern.^8^, ^9^, ^10^, ^11^ Eight cases of post-surgical exogenous panophthalmitis can be found within the literature,^1^^,^^8^^,^^12^, ^13^, ^14^ with two following the implantation of a GDD.^8^^,^^14^

Esporcatte et al. reported a case of rapid-onset panophthalmitis following AGV implantation in a 15-month-old child with primary congenital glaucoma one month after surgery.^8^ The infection was controlled with topical, intravenous and intravitreal antibiotics and tube explantation, although the eye ultimately progressed to phthisis bulbi.^8^ Further, Iqbal et al. reported a delayed-onset panophthalmitis 2 months after surgery in an adult patient following Aurolab Aqueous Drainage Implant (AADI) surgery for refractory glaucoma with Vogt-Koyanagi-Harada (VKH) syndrome. This infection was treated with systemic, topical, and intravitreal antibiotic therapy and evisceration with complete removal of the device. In contrast to the one-month and two-month postoperative onsets described in these reports, our case was characterized by a hyper-acute progression within a few days of surgery, further distinguished by the rapid involvement of peri-orbital soft tissues and necrotizing fasciitis.

Our patient's infection was caused by GAS, also known as *Streptococcus pyogene*s, a gram-positive coccus responsible for a spectrum of diseases ranging from noninvasive conditions such as pharyngitis and cellulitis to more severe infections.^15^ iGAS can be devastating, especially when it progresses to sepsis, streptococcal toxic shock syndrome, or necrotizing fasciitis, which is exceedingly rare when localized to the face and even more uncommon with peri-orbital involvement.^15^, ^16^, ^17^, ^18^ Notably, there was a historic peak in the number of iGAS cases reported in Ontario in January 2024, coinciding with our patient's glaucoma and cataract surgery date.^19^ *Streptococcus pyogenes* is rarely implicated as the causative organism in post-surgical endophthalmitis, with two cases of delayed-onset post-surgical infection described in the literature.^20^^,^^21^

GDD surgery is more susceptible to infection compared to other intraocular procedures such as vitrectomy or cataract surgery given the presence of a communication tract between the inside of the eye and the subconjunctival space via an inserted foreign device.^22^ The precise source and timing of inoculation could not be established. Possible mechanisms include perioperative contamination or early postoperative inoculation, with the GDD potentially serving as a conduit for rapid spread between subconjunctival, intraocular, and orbital tissues,^23^ which may also have contributed to scleral patch dislodgement and further extension of infection.

This case highlights the aggressive nature of iGAS and the complexities of managing panophthalmitis in an elderly patient with multiple comorbidities and metabolic risk factors, particularly long-standing insulin-treated diabetes and hypertension. Our patient carried several potential risk factors for postoperative infection and severe soft tissue infection, including older age, male sex, and long-standing insulin-treated diabetes with microvascular complications.^24^, ^25^, ^26^, ^27^, ^28^, ^29^ Long-standing diabetes is associated with impaired wound healing, which may have adversely affected conjunctival and patch graft healing after AGV implantation.^30^

The fulminant course, positive cultures, and rapidly progressive orbital findings in this case were most consistent with invasive postoperative infection rather than a sterile postoperative inflammatory process. Intravitreal antibiotics were not administered because the patient was critically ill, intubated in the ICU, and had poor visualization in the setting of severe vitreous haze, necessitating prioritization of systemic stabilization and subsequent surgical source control. Patients with systemic comorbidities and impaired host defenses may also be at higher risk of severe iGAS complications, including necrotizing fasciitis, septic shock, and death.^31^

To minimize such complications, we recommend attention to preoperative systemic optimization, including glycemic assessment in patients with diabetes; meticulous sterile technique during device implantation; and a high index of suspicion for infection in any patient with disproportionate pain, fever, confusion, purulent discharge, or rapidly progressive periocular inflammation in the early postoperative period. Early multidisciplinary involvement, including infectious disease, vitreoretinal, and oculoplastics services, may be vision- and life-saving.

This report has limitations inherent to a single-case design. The precise source and timing of inoculation could not be definitively determined, and intraocular microbiologic sampling was not obtained because of the patient's critical systemic condition and rapidly progressive clinical course. Nevertheless, the concordance of the clinical presentation with positive conjunctival and blood cultures for Group A Streptococcus strongly supports fulminant postoperative invasive infection.

## 4 Conclusion

Panophthalmitis is a rare but potentially devastating complication following ocular surgery. This case illustrates the complexity of managing rapidly progressive invasive Group A Streptococcus infection after GDD implantation. Despite timely medical and surgical intervention, the patient developed necrotizing fasciitis, septic shock, and unfortunately passed away due to complications associated with a prolonged medically complex hospitalization. This case underscores the importance of early recognition, multidisciplinary management, and aggressive source control in high-risk postoperative patients.

## CRediT authorship contribution statement

**Armaan Jaffer:** Writing – review & editing, Writing – original draft, Visualization, Project administration, Methodology, Investigation, Data curation, Conceptualization. **Yusuf Ahmed:** Writing – review & editing, Methodology, Investigation, Formal analysis, Data curation, Conceptualization. **Georges Nassrallah:** Writing – review & editing, Supervision, Formal analysis, Data curation, Conceptualization. **Irfan N. Kherani:** Writing – review & editing, Visualization, Supervision, Project administration, Methodology, Investigation, Formal analysis, Data curation, Conceptualization.

## Patient consent

Written consent to publish this case has been obtained.

## Claims of priority statement

After conducting a literature review on July 23, 2025 using PubMed and Google Scholar with the keywords'panophthalmitis,’ ‘necrotizing fasciitis,’ ‘glaucoma drainage devices,’ and ‘exogenous,’ we did not identify prior published reports of exogenous panophthalmitis with peri-orbital necrotizing fasciitis following glaucoma drainage device implantation caused by invasive Group A Streptococcus in an adult patient.

## Authorship

All authors attest that they meet the current ICMJE criteria for Authorship.

## Funding sources

The authors acknowledge support from Toronto Western Hospital and University Health Network in support of this publication.

## Declaration of competing interest

The authors declare that they have no known competing financial interests or personal relationships that could have appeared to influence the work reported in this paper.
